# Differential Dependence on Beclin 1 for the Regulation of Pro-Survival Autophagy by Bcl-2 and Bcl-xL in HCT116 Colorectal Cancer Cells

**DOI:** 10.1371/journal.pone.0008755

**Published:** 2010-01-18

**Authors:** Muriel Priault, Erika Hue, Fanny Marhuenda, Paul Pilet, Lisa Oliver, François M. Vallette

**Affiliations:** 1 CNRS IBGC UMR 5095, Bordeaux, France; 2 Université Victor Ségalen Bordeaux 2, Bordeaux, France; 3 INSERM UMR 892, Nantes, France; 4 Faculté de Médecine, Université de Nantes, Nantes, France; 5 INSERM UMR 791, Nantes, France; Universidade Federal do Rio de Janeiro (UFRJ), Brazil

## Abstract

Autophagy is described to be involved in homeostasis, development and disease, both as a survival and a death process. Its involvement in cell death proceeds from interrelationships with the apoptotic pathway. We focused on survival autophagy and investigated its interplays with the apoptotic machinery. We found that while Mcl-1 remained ineffective, Bcl-2 and Bcl-xL were required for starved cells to display a fully functional autophagic pathway as shown by proteolysis activity and detection of autophagic vesicles. Such pro-autophagic functions of Bcl-2 and Bcl-xL were independent of Bax. However they appeared to operate through non redundant mechanisms as Bcl-xL wielded a tighter control than Bcl-2 over the regulation of autophagy: unlike Bcl-2, Bcl-xL and Atg7 manipulation yielded identical phenotypes suggesting they could be components of the same signalling pathway; Bcl-xL subcellular localisation was modified upon starvation, and importantly Bcl-xL acted independently of Beclin 1. Still an intact BH3-binding site was required for Bcl-xL to stimulate a fully functional autophagic pathway. This study highlights that, in addition to their well-established anti-death function during apoptosis, Bcl-2 and Bcl-xL have a broader role in cell survival. Should Bcl-2 and Bcl-xL stand at the cross-roads between pro-survival and pro-death autophagy, this study introduces the new concept that the regulation of autophagy by Bcl-2 and Bcl-xL is adjusted according to its survival or death outcome.

## Introduction

Macro-autophagy (hereafter referred to as autophagy) is a catabolic process orchestrated by the evolutionary conserved *ATG* genes (for autophagy) [1], [2], and consist in the random sequestration of macromolecules by newly formed double or multiple membrane bound vesicles called autophagosomes. Owing to their size (usually between 500–1500 nm in mammalian cells) [3], [4], autophagosomes can enclose soluble material as well as whole organelles. Degradation of the cargo is achieved after nascent autophagic vacuoles have fused with lysosomes [5]. The resulting products are then available for recycling in biosynthetic pathways; thus autophagy is one of the main lysosomal pathways for biological material turnover.

Autophagy was initially characterised as a survival mechanism since it allows cells to overcome stringent conditions thereby extending the life span. Upon starvation, mutations in *ATG* genes result in cell death in yeast, chlorosis in plants, and decreased adult life span in the *daf-2* Caenorhabditis elegans mutant [1]. In mammals, autophagy exists at a basal level and controls homeostatic functions. Stimulation of autophagy was long known as a response to starvation or hormonal stimulation [6]; however, recent studies have extended the cytoprotective role of autophagy to maintenance of cell viability by showing that *ATG* genes are necessary for survival in different settings in mammals [7]–[10]. Incidentally in these works, autophagy was elegantly shown to be critical for bioenergetics maintenance and cell viability in vitro, but also to play an essential part in vivo in the survival of the whole organism.

Beside this physiological role in tissue homeostasis, autophagy is also paradoxically associated with cell death. This concept arose from the observation that autophagy is commonly seen in dying cells when massive elimination is required in organs [11]. The existence of “autophagic cell death” rather than “cell death with autophagy” [12] was long questioned because autophagy and apoptosis are often activated together in response to stress [13]–[15] although displaying distinct morphologies [16]. Direct evidence of an *ATG*-dependent cell death was brought both by loss of function studies showing that the down-regulation of *ATG7* or *ATG5* could suppress cell death in apoptosis-deficient cells [17], [18], and also by over-expression experiments showing that ectopic expression of mutants of *ATG6*/Beclin 1 could trigger autophagy stimulation which resulted in *ATG5*-dependent cell death [19]. Thus, autophagy can be envisaged as an alternative death program, at least in cells with impaired apoptosis machinery. Yet, if autophagic death is now acknowledged, the causal event for its onset is still unknown as (i) no definitive proof of autophagy as the primary initiator of death has been reported, and (ii) evidence is still lacking of a stimulus that would skew pro-survival autophagy into a deadly program.

More recently, the field of interest for autophagic cell death has widened due to the finding that autophagy deregulation is implicated in cancer, and because molecular interplays have substantiated a crosstalk between apoptosis and autophagy [20]. Hence, the fate of cells is undoubtedly ruled by the coordinated regulation of apoptosis and autophagy, but the when and how are still unresolved questions [21], [22]. One decisive breakthrough as to this latter point was the finding that the key regulator of autophagy Beclin 1 harbours a BH3 domain [23] which binds to the BH1-BH2 domains of anti-apoptotic Bcl-2 [19] or Bcl-xL [24]. Consecutively, thorough analyses of the regulation of autophagy by Bcl-2 and Bcl-xL were undertaken and revealed that their binding to Beclin 1 resulted in the inhibition of autophagy. This binding was initially proposed to divert Beclin 1 from interacting with and stimulating class III phosphatidylinositol-3-kinase Vps34 activity [19], but a consensus now argues against a mutually exclusive interactions of Beclin 1 with Bcl-2 and Vps34 [24]–[26], underlining that autophagic activity is not solely driven by Beclin 1 association with Bcl-2/Bcl-xL. Accordingly, Zeng et al. have reported settings where Beclin 1 does not bind Bcl-xL nor Bcl-2 [27], showing that these interactions are not obligate. All these observations indicate that the functional relevance of Bcl-2/Beclin 1 or Bcl-xL/Beclin 1 interactions is not fully understood yet. Even more, the view of Bcl-2 as an anti-autophagic protein [28]–[30] was challenged by the finding that over-expression of Bcl-2 in apoptosis-deficient cells could instead potentiate autophagy [18].

To further investigate this topic, we decided to explore the autophagic functions of Bcl-2 family members under conditions where autophagy stimulation serves as a survival process: similar to what was observed with death-promoting autophagy, we found that survival autophagy is specifically entangled with anti-apoptotic functions of Bcl-2 and Bcl-xL; however interestingly, they were both found to stimulate autophagy and exhibited different dependence on Beclin 1 to play such pro-survival functions.

## Results

### Starvation-Induced Autophagy Is a Survival Process in HCT116 Cells

**Figure 1:**
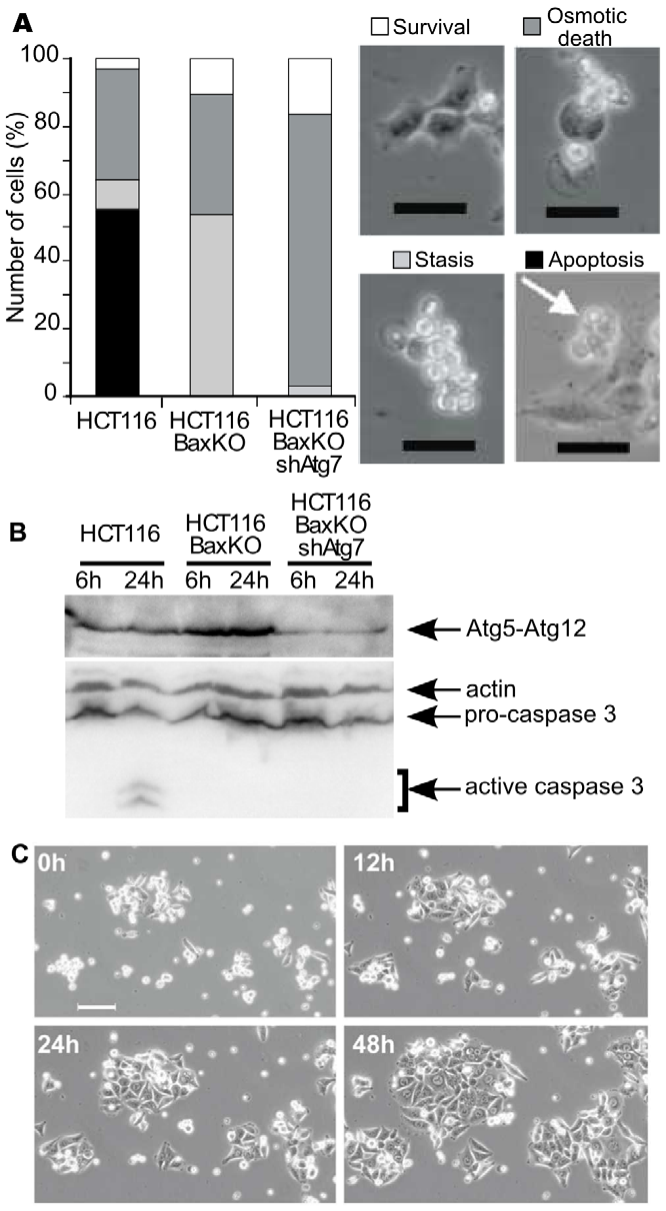
Starvation stimulates survival autophagy in HCT116 cells. (A) Morphometric analyses of cells starved for 48 hours. Cells were plated in complete media and time-lapse video microscopy was started upon transfer into HBSS. Viable cells are defined as adherent cells, apoptotic cells are defined as cells exhibiting multiple membrane blebs, death by loss of osmotic integrity is defined as cells displaying the swelling of a single bleb, and stasis cells are defined as cells that have detached from the substratum but maintain plasma membrane integrity. A minimum of 700 cells were analysed over triplicate experiments. Scale bar: 50 µm. (B) Western blots showing caspase 3 activation and Atg5-Atg12 conjugate in whole cells extracts from cells starved for the indicated times. (C) The non-adherent HCT116-BaxKO cells from 24 hours-starved cultures were collected and transferred into complete medium. Cells were allowed to adhere for 6 hours, then time-lapse video microscopy was started for 48 hours. Scale bar: 100 µm.

The controversy as to the pro-survival [7], [10] or pro-death [17]–[19] outcome of the manipulation of autophagy under nutrient limiting conditions has been fueled by the confrontation of several studies. Therefore we first set out to address the fate of starved cells in our settings: colorectal HCT116 cells were chosen as they appeared, among the all the cell lines we have tested, to exhibit the strongest autophagic response when confronted to nutrient limitation (our unpublished data). Cells were transferred into starvation medium and followed by video microscopy over 48 hours. Morphometric analyses (Fig. 1A) revealed that HCT116 parental cells mostly displayed apoptotic features (∼55%) as confirmed by caspase 3 activation (Fig. 1B) while ∼30% underwent a default death resulting from a rapid osmotic demise (within 20 to 30 minutes after detachment from the plate), and less than 5% remained alive. HCT116-BaxKO subclone was also analysed to assay the outcome of starvation in apoptosis deficient cells: as expected morphometric analysis (Fig. 1A) and western blot (Fig. 1B) failed to detect any apoptotic feature, and we observed that the fraction of cells undergoing the osmotic demise remained unchanged (∼30%). Interestingly, another phenotype predominated as more than 50% of HCT116-BaxKO cells detached from the plate but maintained viability since they recovered normal adherence and proliferation when transferred back into complete medium (Fig. 1C). Of note, these detached cells could not account for mitotic cells after 48 hours of starvation since nutrient limitation is known to induce a rapid cell cycle arrest [10], [31]. To ascertain that this reversible “stasis state” was relevant of the autophagic metabolically quiescent phenotype Lum et al. have described [10], we next assayed the requirement for a functional autophagic machinery. Autophagy uses two ubiquitin-like conjugation systems for autophagosomes completion, which both involve Atg7, a protein reminiscent of the E1 ubiquitin-activating enzymes [32] essential for autophagy [33]. As a result, Atg5 is conjugated to Atg12 when autophagy is activated; western blot revealed that starvation massively induced autophagy in HCT116 BaxKO subclone compared to parental cells (Fig. 1B). Genetic impairment of autophagy was also performed using shRNA targeting of Atg7, and starved HCT116-BaxKO shAtg7 cells were found to undergo massive osmotic cell death (∼80%) due to defective entry into the autophagic stasis state (<5%) (Fig. 1A), as confirmed by the dramatic decrease in Atg5-Atg12 conjugate in these cells (Fig. 1B). The stasis state was thus dependent on autophagy and proved a means to keep death at bay. We conclude that, although transient because cells eventually die if starvation is prolonged, autophagy confers a survival advantage in our settings.

### The Autophagic Capacities of Colorectal HCT116 Cells Is Independent of Bax

**Figure 2:**
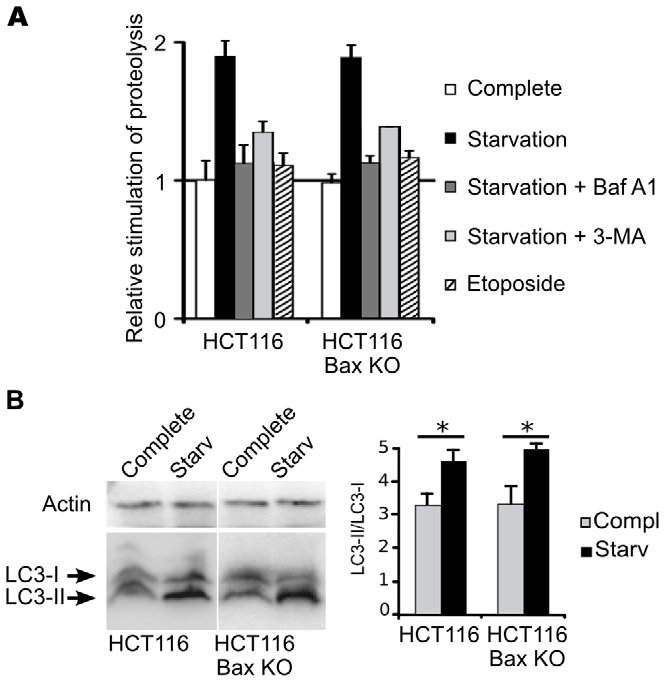
HCT116 and HCT116-BaxKO cells display comparable autophagic capacities. (A) HCT116 and HCT116-BaxKO cells were incubated with L-[^14^C]valine, and chased for 9 hours in complete medium (white bars), HBSS (black bars) or HBSS supplemented with either 0,1 µM Baf A1 (dark gray bars), or 10 mM 3-MA (light gray bars), or in complete medium +20 µM etoposide (hatched bars). Results report the stimulation of proteolysis relative to the respective basal levels measured under non-starving conditions (white bars). The values are the means of at least 3 independent experiments ± s.d. (B) Western blot and quantification of the conversion of LC3-I into phosphatidylethanolamine-conjugated LC3-II. Cells were grown in complete medium or starved for 9 h in the presence of E-64d (20 µg/ml) and leupeptine (20 µg/ml) to prevent the degradation of intra-autophagosomal LC3-II. 150 µg whole cell lysate were loaded on 15% SDS-PAGE. *Student test p<0,02.

The previous experiment suggested that when cells can execute both apoptosis and autophagy, apoptotic phenotypes prevail over autophagic features. We were therefore concerned by the possibility that the autophagic response could indeed be impaired by the execution of apoptosis. To address this question, we have compared the autophagic capacity of HCT116 and HCT116-BaxKO cells under conditions where macroautophagy is the predominant form of protein degradation, namely after 6–9 hours of starvation [34]. Fig. 2A shows the degradation of L-[^14^C]valine-labeled long-lived proteins. The basal proteolysis measured under control conditions was comparable in both cell lines and respectively set as the internal reference (white bars). Starvation similarly resulted in a two-fold increased proteolysis in both cell lines (black bars). This proteolysis was (i) essentially lysosomal since Bafilomycin A1 (Baf A1, an inhibitor of lysosomal ATPase proton pump) completely prevented its augmentation (dark gray bars), and (ii) associated with autophagy since its stimulation was significantly reversed by 3-MA (light gray bars). As a control, an apoptotic induction by etoposide over the same period of time did not stimulate any proteolysis (hatched bars). We also assayed the conversion of cytosolic Atg8/LC3-I into the autophagosome-bound phosphatidylethanolamine conjugate LC3-II, which is one of the hallmarks of autophagy. Western blots (Fig. 2B) confirmed that HCT116 and HCT116-BaxKO both produced comparable amounts of LC3-II upon starvation. The diffuse cytosolic of punctate localisation of mCherry-LC3 was also monitored in mouse embryonic fibroblasts (MEFs) as an alternative cell line, and showed that starvation triggered the same autophagosomal relocalisation of LC3 in wild-type MEFs and Bax knocked-out MEFs (Fig. S1). Taken together, our experiments show that apoptosis execution does not impair the autophagic response, and we conclude that the autophagic capacity is independent of Bax.

### Ectopic Expression of Bcl-2 and Bcl-xL, but Not Mcl-1, Stimulates Autophagy

**Figure 3:**
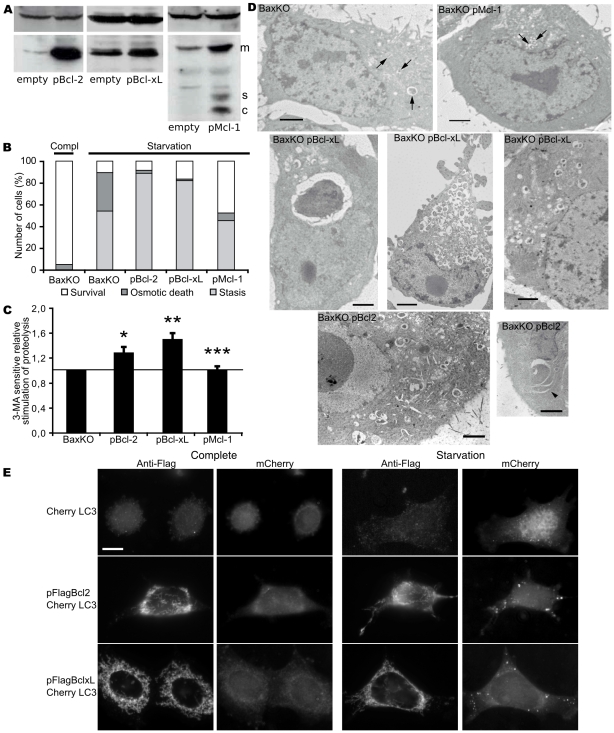
Bcl-2 and Bcl-xL stimulate survival autophagy while Mcl-1 does not. (A) Western blots of cells transfected with an empty vector, or vectors encoding Bcl-2 or Bcl-xL or Mcl-1. 50 µg of protein extracts were analysed on a 12% SDS-PAGE. Mcl-1 was detected as a mature protein (m), a spliced variant (s) and a caspase-cleaved product (c). (B) Morphometric analyses of HCT116-BaxKO-derived stable cell lines: cells were plated in complete media and time-lapse video microscopy started when cells were transferred to HBSS (starvation) or not (complete). A minimum of 700 cells were analysed in at least triplicate experiments. (C) Relative stimulation of starvation-induced 3-MA-sensitive degradation of long-lived proteins in HCT116-BaxKO cell lines transfected or not with Bcl-2 or Bcl-xL or Mcl-1. Cells were chased for 6 hours in HBSS or HBSS + 3-MA. The 3-MA sensitive activity measured in HCT116-BaxKO cells was set at 1 and results represent the 3-MA sensitive activities of each cell line relative to that found in untransfected cells. Data are the mean (±s.d.) of at least 3 independent experiments. Student test was used for statistics significance of the results compared to untransfected cells: (*)p = 0,010 (**)p = 0,001 and (***)p = 0,479. (D) TEM of HCT116-BaxKO cells over-expressing Bcl-2 or Bcl-xL or Mcl-1 after 6 hours of starvation in HBSS. Arrows point at degradative autophagosomes. Scale bar: 2 µm. (E) 24 h after transfection either with mCherryLC3 alone, or with mCherryLC3 and Flag-Bcl-xL or Flag-Bcl-2, BaxKO MEF cells grown in complete medium or starved for 6 hours were fixed. Immonocytochemistry reveals Flag-tagged constructs (green). Pictures are representative of at least 4 independent experiments. Scale bar: 10 µm.

The latter result provided grounding for our resolution to use the apoptosis-deficient subclone HCT116-BaxKO to address the regulation of pro-survival autophagy by anti-apoptotic proteins, while separating the contribution of the Bcl-2 family members to apoptosis. HCT116-BaxKO cells were transfected with plasmids encoding either Bcl-2, Bcl-xL or Mcl-1, resulting respectively in 9, 2 and 3 fold protein increase (Fig. 3A). Starvation did not modify the expression profiles (Fig. S2). Video microscopy of starved cells showed that over-expression of Bcl-2 and Bcl-xL significantly increased the number of cells entering the autophagic stasis state (Fig. 3B, respectively 88% and 82%), whereas Mcl-1 over-expression augmented instead the number of viable adherent cells (Fig. 3B, white bar  = 48%). Thus, Bcl-2 and Bcl-xL clearly had a different effect than Mcl-1 on autophagy. This was confirmed by autophagic proteolysis (Fig. 3C) and transmission electron micrographs (TEM) wherein degradative vesicles were more abundant in cells expressing Bcl-2 and Bcl-xL while those expressing Mcl-1 remained as untransfected cells (Fig. 3D, and Fig. S3 for magnification); of note, occasionally, extensive vacuolisation appeared in the cytoplasm of cells over-expressing Bcl-xL (Fig. 3D, left panel). When we used BaxKO MEFs and monitored mCherry-LC3 fluorescence pattern, we found that the relocalisation of the protein toward punctate structures was significantly stimulated in Bcl-2 or Bcl-xL transfected cells (Fig. 3E), thus showing that the phenomenon was conserved in an alternative cell line. All the gold standard techniques used so far unanimously showed that only autophagic vesicles (AVs) are affected by Bcl-2 or Bcl-xL over-expression while other lipidic vesicles like multi-vesicular bodies are not; therefore we used monodansylpentane (MDH) [35] (a lipophilic dye which already proved to stain AVs [36]) to run computer-assisted analyses of AVs, measure their size, and determine their frequency of detection (Fig. S4): starved HCT116-BaxKO cells transfected with Bcl-2 or Bcl-xL revealed an augmentation of the number of AVs, correlated with an increased frequency of appearance of larger AVs, some being twice as big as classical autophagosomes as confirmed by occasional TEM. On the opposite, Mcl-1 transfected cells were not statistically different from untransfected cells. Hence we conclude that, while Mcl-1 does not play an essential role in survival autophagy, Bcl-2 and Bcl-xL specifically stimulate the autophagic capacity and increase the AVs number and size.

### Down-Regulation of Bcl-xL Is a More Potent Inhibitor of Autophagy Than That of Bcl-2

**Figure 4:**
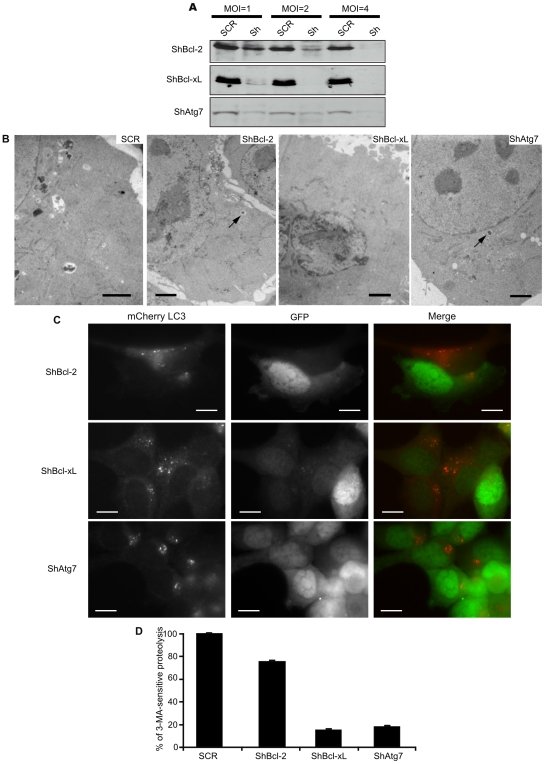
Silencing of Bcl-2 and Bcl-xL has non-redundant effects on autophagy. (A) Western blots of HCT116-BaxKO cells infected with increasing amounts of viral particles transducing shRNA against Bcl-2, Bcl-xL or Atg7, or a scrambled shRNA (SCR). Total protein extracts were performed, and 50 µg proteins were analysed on SDS-PAGE. (B) HCT116-BaxKO cells infected with viral particles carrying shRNA against Bcl-2, Bcl-xL or Atg7 with a MOI = 4 were analysed for autophagic degradation of long-lived proteins shortly after infection. Cells were chased for 6 hours in HBSS or HBSS+10 mM 3-MA. 3-MA sensitive activity measured in SCR-infected cells was set to 100%, and results represent the 3-MA sensitive activities of each infected cell line relative to that found in SCR-infected cells. Data are the mean (±s.d.) of at least 3 independent experiments. When the error is not indicated, bar was too small to be seen. (C) HCT116-BaxKO cells stably transfected with mCherryLC3 were infected with viral particles carrying shRNA against Bcl-2, Bcl-xL or Atg7 with a MOI = 4. Cells were analysed for their GFP fluorescence (infected cells) and LC3 relocalisation after 6 hours of starvation. Scale bar: 15 µm. (D) TEM after 6 hours of starvation of HCT116-BaxKO cells where Bcl-2 or Bcl-xL or Atg7 expression was silenced. Scale bar: 2 µm.

We next explored the effect of Bcl-2 and Bcl-xL down-regulation as compared to that of Atg7 in starved cells. A multiplicity of infection (MOI) of 4 caused at least a 90% silencing of the target genes (Fig. 4A) without any cross effect on each other (not shown). Over the time frame of the experiment, viability of transduced cell remained at control values (>90%, data not shown) both in complete medium or after the 6h starvation. TEM showed that double membrane bound vesicles were barely detectable in starved cells wherein Bcl-2, Bcl-xL or Atg7 had been down-regulated as compared to SCR transduced cells (Fig. 4B). The plasmid used to transduce the shRNAs encodes the green fluorescent protein (GFP) and we could thus correlate the efficiency of infection with the autophagic response revealed by mCherryLC3 localisation (Fig. 4C). Upon starvation, cells displaying a high degree of transduction with shAtg7 (highly GFP-positive cells) exhibited a diffuse cytosolic distribution of mCherryLC3 showing that autophagy was efficiently impaired, while non transduced cells displayed punctate mCherryLC3 organisation. In keeping, starved cells wherein shBcl-2 or shBcl-xL had been efficiently transduced exhibited a diffuse mCherryLC3 staining, although shBcl-xL reproducibly triggered a stronger inhibitory phenotype than shBcl-2. Thus, Bcl-2 or Bcl-xL down-regulation had an inhibitory effect on autophagy, however this experiment could indicate that Bcl-2 and Bcl-xL may not be completely redundant for the molecular control of survival autophagy. In an attempt to quantify such a difference, we finally assayed starvation-induced proteolysis of shRNAs transduced cells: for a MOI of 4, Bcl-2 knocked-down cells retained 75% of the 3-MA sensitive proteolysis as compared to a scramble shRNA (SCR). This plateau was already reached with a MOI of 2 and remained unchanged even for higher MOIs (not shown). In contrast, Bcl-xL and Atg7 silencing decreased the degradation rate respectively to 15 and 17% (Fig. 4D). Hence, Bcl-2 and Bcl-xL knock-down both had a compelling inhibitory effect on autophagic proteolysis. However, only Bcl-xL down-regulation mimicked that of Atg7 and proved a key molecular control of autophagy.

### Bcl-xL Regulates Survival Autophagy Independently of Beclin 1

Studies reporting anti-autophagic functions of Bcl-2 and Bcl-xL depict conditions where stimulated autophagy resulted in cell death, and showed that their binding to Beclin 1 blocked the onset of autophagy. On the opposite, Zeng et al. reported that in non-starved U-251 glioblastoma cells, neither Bcl-2 nor Bcl-xL were normal endogenous binding entities for Beclin 1 [27], suggesting that depending on the settings, these proteins are not obligate partners.

**Figure 5:**
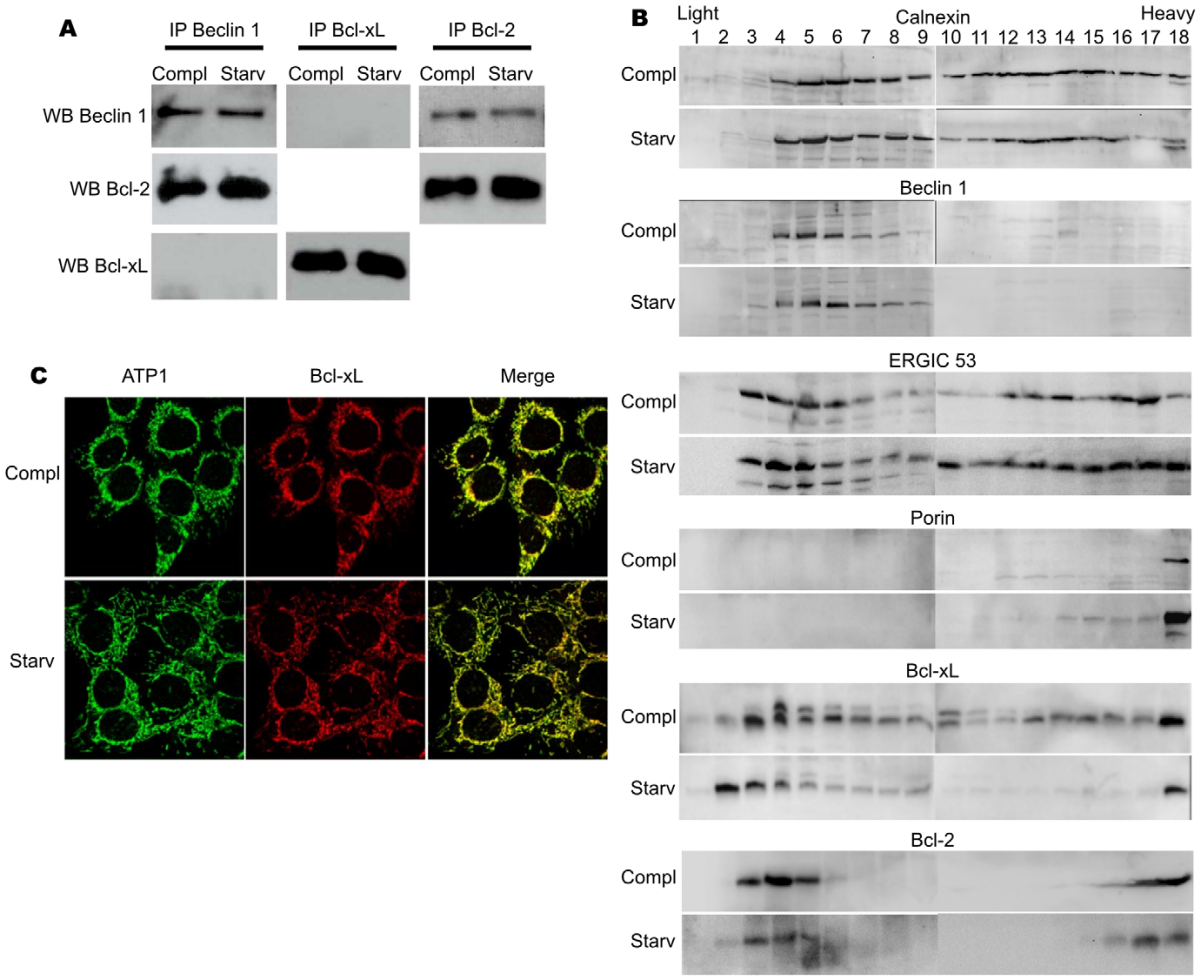
Differences between Bcl-2 and Bcl-xL behaviour during autophagy. (A) Co-immunoprecipitation of endogenous Beclin 1 with endogenous Bcl-2 and Bcl-xL in HCT116-Bax KO cells grown in complete medium or starved for 6 hours. (B) Subcellular fractionation of HCT116-Bax KO cells grown in complete medium or starved for 6 hours. Cells were broken with a Dounce homogeniser, post-nuclear supernatants were loaded on top of a continuous 10–55% sucrose gradient for ultracentrifugation overnight. Fractions of 500 µl were collected, and total protein precipitated. The same volume of each fraction was separated on a 14% tris-tricine SDS-PAGE. Western-blots were as described in the methods section. (C) Immunocytochemistry on endogenous Bcl-xL and ATP1 in HCT116-BaxKO cells grown in complete medium or starved for 6 hours.

In our paradigm of cytoprotective autophagy, endogenous Beclin 1 was immunoprecipitated from control or starved cell lysates (Fig. 5A). Bcl-2 was efficiently pulled down but not Bcl-xL; of note, Bcl-2/Beclin 1 association did not vary in starved versus control cells. The converse immunoprecipitation of endogenous Bcl-2 confirmed these observations, while Bcl-xL again failed to pull-down any detectable Beclin 1. These experiments support a differential regulation of pro-survival autophagy by Bcl-2 and Bcl-xL, but further experiments were needed to ascertain that Bcl-xL acted independently of Beclin 1. Subcellular fractionation showed that excepting the mitochondrial portion of Bcl-2, substantial amounts of Bcl-2 and Beclin 1 overlap in light fractions, and in further keeping with IP results, none of these proteins changed localisation in control or starved cells. In contrast, Bcl-xL was detected throughout the whole gradient in control cells and was relocalised to very light fractions in starved cells; given the number of compartments in which Bcl-xL is present, it is reasonable to assume that it may interact with many partners, and this may account for the fact that Beclin 1 is not found as its main interacting partner through IP experiments. Confocal analyses showed that Bcl-xL co-localised with the mitochondrial inner membrane protein Atp1 in control and starved cells (Fig. 5C); hence the light fractions hosting Bcl-xL in starved cells are in the close vicinity of mitochondria.

**Figure 6:**
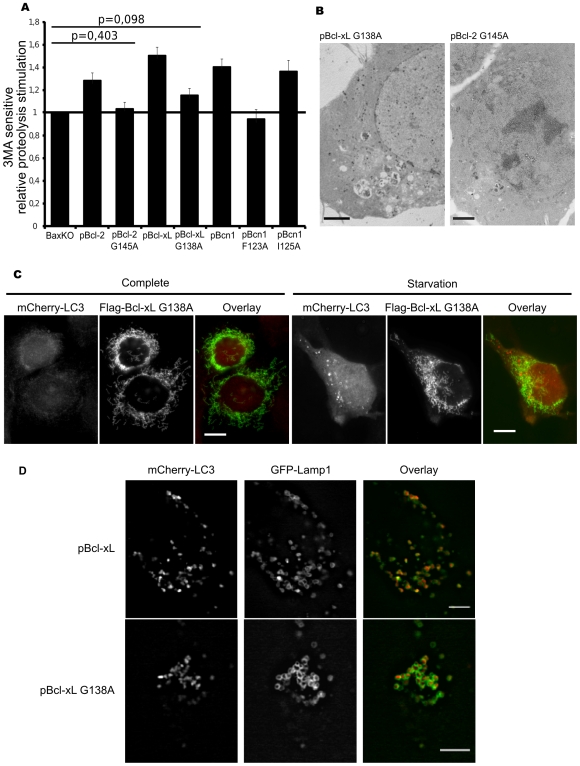
Requirement for an intact BH3 binding site for Bcl-2 and Bcl-xL pro-autophagic functions. (A) Relative stimulation of starvation-induced 3-MA-sensitive degradation of long-lived proteins in HCT116-BaxKO cell lines transfected or not with Bcl-2, Bcl-2^G145A^, Bcl-xL, Bcl-xL^G138A^, Beclin 1, Beclin 1^F123A^ or Beclin 1^I125A^. Cells were chased for 6 hours in HBSS or HBSS + 3-MA. Results represent the 3-MA sensitive activities of each cell line relative to that found in untransfected cells. Data are the mean (±s.d.) of at least 3 independent experiments. Student test was used for statistics significance of the results compared to untransfected cells; p values not indicated on the graph are p<0,01. (B) TEM of HCT116-BaxKO cells transfected with Bcl-xL^G138A^ (left) or Bcl-2^G145A^ (right) after 6 hours of starvation. Scale bar: 2 µm. (C) 24 h after cotransfection of mCherryLC3 with Flag-Bcl-xL^G138A^, BaxKO MEFs grown in complete medium or starved for 6 hours were fixed. Immonocytochemistry reveals Flag-tagged constructs (green). Pictures are representative of at least 4 independent experiments. Scale bar: 10 µm. (D) HCT116BaxKO cells over-expressing Bcl-xL or Bcl-xL^G138A^ were transfected with mCherryLC3 and mEGFP-Lamp1A. After 6 h of starvation, cells were fixed and observed with a 100x objective (scale bar: 5 µm).

Because IP experiments showed an interaction between Bcl-2 and Beclin 1 but were inconclusive for Bcl-xL and Beclin 1, we finally decided to set up a last array of experiments to further explore the functional differences between Bcl-2 and Bcl-xL and their dependence on Beclin 1. We analysed autophagy in cells over-expressing Beclin 1 or the BH3 mutants Beclin 1^F123A^ and Beclin 1^125A^ which respectively loose or retain their binding to Bcl-2 and Bcl-xL[19], [37] (protein expression levels are shown in Fig. S5). Fig.6A shows that, as expected, Beclin 1 ectopic expression stimulated starvation-induced autophagic proteolysis. Such a trait was strictly dependent on a functional BH3 domain (i.e. able to interact with BH1-BH2 containing partners) since Beclin 1^125A^ mimicked the wild type protein, while Beclin 1^F123A^ was completely unable to trigger autophagy. The BH1 mutants of Bcl-2 and Bcl-xL (i.e. Bcl-2^G145A^ or Bcl-xL^G138A^), which both loose their interaction with the BH3 domain of Beclin 1 [19], [24] were reciprocally analysed. Levels of over-expression in HCT116-BaxKO cells were similar to those of ectopic Bcl-2 and Bcl-xL (Fig. S2). Preventing the binding of Bcl-2 to Beclin 1 completely abrogated the stimulation of autophagic proteolysis observed with native Bcl-2. TEM confirmed that the amount and size of autophagic vesicles in Bcl-2^G145A^ expressing cells were very different from Bcl-2 expressing cells, and were indeed comparable to non transfected cells (compare Fig. 3D and Fig. 6B right). Therefore, Bcl-2 pro-autophagic functions entirely rely on the binding of a BH3 containing partner (probably Beclin 1 according to IP experiments and to proteolysis data obtained with Beclin 1^F123A^). In contrast, Bcl-xL^G138A^ still retained a slight capacity to stimulate autophagic degradation, although it was far less efficient than its native counterpart (Fig. 6A). TEM indicated that Bcl-xL^G138A^ stimulated AVs synthesis as efficiently as Bcl-xL (compare Fig. 6B left with Fig. 3D). Relocalisation of mCherry-LC3 in BaxKO-MEFs transfected with Bcl-xL^G138A^ confirmed this observation (compare Fig. 6C with Fig. 3E). Colocalisation of LC3 and Lamp1A in starved HCT116 BaxKO cells expressing Bcl-xL or Bcl-xL^G138A^ showed that autophagic vesicles contained both markers, and hence represent degradative autophagosomes. Therefore, the mutation of Bcl-xL BH3 binding site resulted in a structurally intact but functionally impaired autophagic pathway. This set of data indicate that degradative AVs are formed in Bcl-xL^G138A^ but further experiments are needed to explain why these AVs are less functional than in Bcl-xL expressing cells. Comparison of the amount of Atg5-Atg12 conjugate in non transfected cells or in cells expressing Bcl-xL or Bcl-xL^G138A^ failed to identify any difference, indicating that the conjugation step is not limiting and cannot be further stimulated by Bcl-xL expression (Fig. S6). Future experiments will aim at comparing the kinetics of AVs maturation in Bcl-xL and Bcl-xL^G138A^ expressing cells. As a conclusion, Bcl-2 and Bcl-xL exhibit a differential dependence on Beclin 1 do display their pro-autophagic functions. While Bcl-2 fully relies on its binding to Beclin 1, Bcl-xL does not control the formation of autophagosomes via Beclin 1, and we propose that a protein which uses the BH3-binding domain helps Bcl-xL stimulating a functional autophagic pathway.

## Discussion

The molecular bases to predict the cytoprotective or the pro-death outcome of autophagy are still a topic of intense investigation as the execution of the former or the latter drives cells toward opposite fate. Addressing the interplays between apoptosis and autophagy was one way to approach this issue. Settings in which the stimulation of autophagy results in autophagic cell death [19] have pointed at direct interactions between Beclin-1 and Bcl-2 or Bcl-xL as potential convergence point for the two death processes that are apoptosis and autophagy. We resolved to study the converse regulation of pro-survival autophagy by Bcl-2 family members. A wide discrepancy has been reported in the autophagic response of cancer cells [20], and colorectal HCT116 cells were chosen for this study as highly responsive cells when confronted to starvation (our unpublished data). Our settings delineated a “stasis state” strictly correlated to autophagy, and which proved to be a reversible quiescent phenotype allowing cells to sustain viability during nutrient restriction (Fig. 1). Such cytoprotective autophagy appeared independent of Bax (Figs 2, S1), which allowed us to choose the apoptosis-deficient subclone HCT116-BaxKO to investigate the autophagic functions of Bcl-2 family members while circumventing their commitment to apoptosis regulation. Cytoprotective autophagy appeared to be specifically regulated by Bcl-2 and Bcl-xL, not by Mcl-1. Opposite to autophagic cell death, survival autophagy was stimulated by Bcl-2 and Bcl-xL, as unanimously shown by proteolysis experiments, TEM and LC3 re-localisation assays based on over-expression and silencing of the two proteins. Bcl-2 and Bcl-xL also seemed committed in the regulation of AVs synthesis, but apparently through non redundant mechanisms (Fig. 4, 5, 6). Such observations are in keeping with Shimizu et al., who already suggested possible functional differences between Bcl-2 and Bcl-xL for the stimulation of autophagy [18]. We found that, indeed, Bcl-xL wielded a tighter control than Bcl-2 over AVs production (Fig. 4), and gathered evidence that Bcl-xL and Atg7 could belong to the same pathway since the down-regulation of the former phenocopied that of the latter (Fig. 4B, C). More importantly, in this pathway Bcl-xL did not control autophagy via Beclin 1 (Figs. 5 & 6).

Beclin 1 was reported to be engaged in different interactions depending on the settings: the mammalian class III PI3-kinase hVPS34 is documented as its major partner [27], [38], this interaction resulting in the stimulation of VPS34 by Beclin 1. While the function of this tandem remains to be elucidated under nutrient rich conditions [27], its is known to be essential in the earliest steps of autophagosomes biogenesis during autophagy. Pattingre et al. proposed that nutrient-driven regulation of Beclin 1/hVPS34 interaction was mediated by Bcl-2: when nutrients are abundant, Bcl-2/Beclin 1 interaction would be stabilised at the expense of Beclin 1/hVPS34 binding [19]. At odds with this scheme, under nutrient rich conditions Zeng et al. never detected any Bcl-2/Beclin 1 or Bcl-xL/Beclin 1 interactions [27], and other studies showed that Beclin 1/hVPS34 and Bcl-2/Beclin 1 are non mutually exclusive interactions [24]–[26]. In our settings, we found that Bcl-2 interacted with Beclin 1: this interaction remained unchanged by autophagy-promoting conditions (Fig. 5A) and was absolutely required for Bcl-2 pro-autophagic functions (Fig. 6). Maiuri et al showed that interactions between Beclin 1 and mitochondrial Bcl-2 remained constant [24] while interactions with ER-located Bcl-2 did not. In our settings, non mitochondrial Bcl-2 can hardly account for an ER localisation (Fig. 5B); further experiments are required to identify the host compartment and characterise the molecular basis for such pro-autophagic functions.

Conversely, Bcl-xL did not interact with Beclin 1 in our model, and accordingly BH3-binding defective mutant Bcl-xL^G138A^ stimulated AVs formation as efficiently as native Bcl-xL (Fig. 3 & 6), suggesting Beclin 1-mediated AVs formation was not affected by this mutation. However, we observed that Bcl-xL^G138A^ was less efficient than Bcl-xL to stimulate autophagic proteolysis, and therefore we cannot rule out the possibility that unknown functions of Beclin 1 are yet to unveil, which would control steps downstream of autophagosomal membrane nucleation, and would depend on Bcl-xL binding. Our results suggest that both functions would be separable since, opposite to the supposed alternative function of Beclin 1, the formation of AVs is never impaired in our model, providing a unique tool to address how Bcl-xL would control it. An alternative explanation is that another protein, as yet unidentified, is required for autophagosomes formation, and activated by the binding of Bcl-xL, the interaction site encompassing the BH3 binding domain of Bcl-xL.

The array of functions a given protein can wield is strictly dependent on its subcellular localisation. A major unsolved question when considering the autophagic functions of Bcl-2 and Bcl-xL was their respective localisation: Beclin 1 and hVPS34 are reported to localise at the trans-golgi network, while enforced ER-targeted Bcl-2 and Bcl-xL were reported to regulate autophagy [19], [24]. As observed by Pattingre et al., we confirmed that the localisation of Beclin 1 did not vary depending on the cellular nutrient status [19], but we found that in autophagic cells Bcl-xL re-localised to lighter membrane fractions. So did Bcl-xL^G138A^ (not shown). These observations may stand as another clue of a Beclin 1-independent regulation of autophagy by Bcl-xL, and widen the array of partners available to account for Bcl-xL autophagic functions. In light of our data, we subscribe to the proposition by Luo and Rubinsztein who recently suggested that by virtue of their interaction with BH3 containing proteins, Bcl-2/Bcl-xL may have pro- or anti-autophagic functions [39]. Identification of Bcl-xL binding partners under autophagic conditions is currently under scrutiny in our laboratory.

To conclude, we found Bcl-2 and Bcl-xL stimulate survival autophagy, and help the formation of autophagosomes through non redundant mechanisms, Bcl-xL acting on the whole independently of Beclin 1. When confronted to the literature, our set of data supports the view that the regulation of autophagy by Bcl-2 and Bcl-xL would be adjusted according to the survival or death outcome of autophagy. Both pro- and anti-autophagic functions have been attributed to Bcl-2 and Bcl-xL, the former being related to cytoprotective autophagy, and the latter to autophagy which leads to cell death. Remarkably, Bcl-2 and Bcl-xL always retain pro-survival functions. Hence, these molecules exceed the well-established anti-death functions they display during apoptosis. Their role in the differential regulation of autophagy, either as a cytoprotective mechanism or as a death process, is still of particular interest with respect to cancer.

## Materials and Methods

### Materials

All cell culture material was obtained from Gibco (Invitrogen). Unless stated otherwise, all chemicals were from Sigma. The plasmids encoding mCherry-LC3 and monomeric EGFP-Lamp1A were respectively kindly given by Pr T. Johansen [40] and Dr E. Dell'Angelica [41]. Flag-tagged constructs were a kind gift of Dr P. Juin.

### Cell Lines and Cell Culture

HCT116 and HCT116-Bax KO were obtained from Dr B. Vogelstein (Baltimore, USA). SV40 immortalised MEFs and Bax/Bak double knocked-out MEFs were obtained from Dr. S Korsmeyer.

HCT116 cells were grown in McCoy's 5A. HEK293FT cells and MEFs were grown in DMEM containing 4,5 g/L glucose. All growth media were supplemented with 2 mM L-glutamine, penicillin (100 U/mL), streptomycin (100 µg/mL) and 10% foetal calf serum (FCS).

Stable cell lines were established from HCT116-BaxKO cells transfected with pRc-CMV or pRc-CMV encoding Bcl-2, Bcl-xL, Bcl-xL^G138A^ or Mcl-1. Cells (1×10^6^) were transfected with 10 µg plasmid DNA using the Gene pulser II (Biorad) and two pulses of 250 V-850 µF. Stable cell lines were established by neomycin (1 mg/mL) selection.

MEFs were transiently transfected using Fugene HD (Roche).

HIV-1 lentivirus-based vectors were used to introduce shRNAs into HCT116-BaxKO cells. ShRNAs were cloned under the control of U6 promoter between Bam-H1 and Hind-III sites in pSilencer 2.1 (Ambion), according to the manufacturer's protocole. PCR amplified fragments were further subcloned between Xba-I and Xho-I sites of FG12 lentivector [42]. HEK293FT cells were used as packaging cells, and virus production was as previously described [42]. Human Bcl-2 siRNA, 5′- CCG GGA GAU AGU GAU GAA G –3′, Bcl-xL siRNA, 5′- AGG AUA CAG CUG GAG UCA G -3′ and Atg7 siRNA, 5′- AGG ATA CAG CTG GAG TCA G -3′ were used.

### Autophagy Assays

Autophagy was induced by amino acids and serum starvation: cells were washed three times with PBS and incubated for 6 to 9 hours in Hank's Buffered Salts Solution (HBSS) buffered with 2,2 g/L NaHCO_3_ and supplemented with 0,1% BSA.

The degradation of radio-active L-[^14^C]valine-labeled long-lived proteins was measured as follows: cells were incubated for 24 hours in complete medium with 0,1 µCi L-[^14^C]valine to label total proteins. Radio-activity was further pre-chased for 1 hour in complete medium in the presence of an excess of L-valine (10 mM) to remove the contribution of short-lived protein degradation. Finally, cells were incubated for 6 to 9 hours either in complete medium or in HBSS in the presence or in the absence of 3-MA and with an excess of L-valine. Supernatants were collected and free amino acids precipitated with 80% trichloroacetic acid (TCA), while proteins in adherent cells were precipitated with 10% TCA. Radio-activity was quantified in a scintillation liquid analyser Tri-carb 2100TR (Packard). Proteolysis is expressed as the percentage of free radio-activity released in the supernatant relative to the total radio-activity.

TEM analyses of autophagy were done on fixed cells with 4% glutaraldehyde in PBS (pH 7,4), followed by 2% OsO_4_ post-fixation. After dehydration in a graded series of ethanol, adherent cells were embedded in Epoxy resin, and thin sections (60 to 70 nm) were cut on a Reichert Ultracut E microtome and stained with uranyl acetate and lead citrate for observation at 80 KV under a JEM-1010 transmission electron microscope (JEOL).

### Time-Lapse Analysis

Time-lapse video microscopy experiments were performed using a Zeiss Axiovert 200-M inverted microscope and the AxioVision 4.6 program. Dishes were placed inside an Incubator XL-3, on a heating insert M06 (37°C) topped with a CO_2_-cover HM connected to a CO_2_ controller that maintained environmental CO_2_ concentration at 5% for the duration of filming. Digital pictures were acquired and saved every 10 minutes over 48 hours using an AxioCam MRm digital camera. The series of photographs were displayed as continuous time-lapse movies for analyses.

### Western Blot

Total proteins were extracted in 1% NP-40, 0,5% sodium-deoxycholate, 0,1% SDS supplemented with protease inhibitor Mini® from Roche Diagnostics. Protein concentration was determined using BCA kit (Interchim). Protein extracts were separated on SDS-PAGE or tris-tricine SDS-PAGE [43], transferred onto PVDF membrane (Millipore) and revealed with ECL (Roche Diagnostics). Primary antibodies were used at 1/1000 dilution: mouse monoclonal anti-actin (Chemicon), rabbit polyclonal anti-Atg5 (Sigma), rabbit polyclonal anti-Atg7 (Rockland), mouse monoclonal anti-Bcl-2 (DakoCytomation), rabbit polyclonal anti-Bcl-x (Transduction Lab), rabbit polyclonal anti-calnexin (Abcam), rabbit monoclonal anti-caspase 3 (Abcam), rabbit polyclonal anti-LC3 (Cell Signaling), rabbit polyclonal anti-Mcl-1 (Pharmingen), rabbit polyclonal anti-ERGIC-53 (Sigma), mouse monoclonal anti-Flag (Sigma) and mouse monoclonal anti-porin (Mitoscience LLC). Horseradish-peroxidase-conjugated secondary antibodies were from Biorad. Quantifications were performed with the software ImageJ.

### Immunoprecipitation

Cells were resuspended in CHAPS buffer (1% CHAPS, 10 mM HEPES, 150 mM NaCl, supplemented with protease inhibitors) then disrupted by 3 cycles of freeze/thawing on ice. Cell debris was removed by centrifugation (20 minutes at 13000 x g). Immunoprecipitation was performed on 500 µg proteins, using a Protein G-agarose Immunoprecipitation Kit (Sigma), and 2 µg antibodies (mouse monoclonal anti-Beclin 1), rabbit monoclonal anti-Bcl-2 (Epitomics), or rabbit monoclonal anti-Bcl-xL (Epitomics). After elution, 1/5 of the eluted proteins were separated on a 12% SDS-PAGE, and Western blot were as described above.

### Immunocytochemistry

Cells were grown on gelatine-coated cover-slips. Cells were fixed in 4% paraformaldehyde for 40 minutes, permeabilised with 0,1% SDS for 10 minutes, blocked with 3% BSA for 20 minutes, and then incubated with primary antibodies for 1 hour followed by secondary Alexa antibodies (Molecular Probes) for 30 minutes. Cells were finally mounted either with Mowiol or Prolong antifade (Molecular Probes, Invitrogen) polymerising solution, and observed under a confocal microscope (LEICA TCS-SP1). Primary antibodies were: mouse monoclonal anti-ATP1 (Molecular probes), rabbit polyclonal anti-Bcl-X (Transduction Lab) and anti-Flag antibody (Sigma).

### Fluorescence Analysis

Images were acquired with an inverted microscope Olympus IX81 CellR imaging system (100x objective). Stacks of 260 nm step were acquired and images were deconvolved with iterative deconvolution 3D plugins from Image J.

## Supporting Information

Figure S1 Mouse embryonic fibroblasts wild type (SV40) or (b) knocked-out for Bax (BaxKO) were transiently transfected using Fugene HD (Roche) with mCherryLC3. Cells were grown on gelatine-coated cover-slips, fixed in 4% paraformaldehyde for 40 mins, and washed. Cells were finally mounted with ProlongTM antifade polymerizing solution (Molecular Probes, Invitrogen) and observed on a Leica epifluorescence microscope.
(0.45 MB TIF)

Figure S2 Stable HCT116 BaxKO cells expressing the indicated proteins of the Bcl-2 family were either grown in complete medium or starved for 6 or 24 h. Whole cell extracts were performed and 100 µg of proteins were separated by SDS-PAGE. Western blot was followed by immunodetection with actin as loading control or the indicated antibodies.
(0.36 MB TIF)

Figure S3 2x zoom of TEM presented in Fig. 3.
(1.82 MB PDF)

Figure S4 Stable HCT116 BaxKO cells expressing the indicated Bcl-2 family proteins were grown on gelatin-coated glass cover-slips to approximately 70% confluence either in complete medium or starved for 6 h. Cover-slips were washed with PBS, and incubated for 30 minutes with 200 µM MDH at 37°C in the dark then washed with PBS, and mounted for immediate observation under UV (λex = 359 nm) on a Leica DMLB microscope. Digital pictures were acquired with a Leica DC 300-F camera. Analyses of the images were done with a home-made program on a Leica Q550 imaging workstation. Computer-assisted analyses were performed on 200 to 400 cells to determine the diameter of all MDH-stained structures. The distribution of the diameters was plotted by intervals of 0,2 µm, and the frequency of occupation of these intervals labeled (% events). A Student test was applied for statistic analysis: in transfected cells, distributions under control conditions were not statistically different from that of untransfected cells (p = 0.42 for pBcl-2 cells, p = 0.24 for pBcl-xL cells, p = 0.2 for Bcl-xLG138A cells, and p = 0.17 for pMcl-1 cells). Within each cell line, the distribution under starved conditions was statistically different from their respective control distribution (p<0.0001) except for Mcl-1 (p<0.4). Within each cell line, distributions under starved conditions were statistically different from starved parental cells (p<0,00005).
(0.78 MB TIF)

Figure S5 HCT116 BaxKO cells were transfected with plasmids encoding either Beclin 1 or the mutants Beclin 1 F123A or I125A. Whole cell extracts were performed and 100 µg of proteins were separated on SDS-PAGE. Western blot was followed by immunodetection of Beclin 1. Quantification with Image J software indicated that compared to endogenous level, the overexpression levels were 1.7 in transfected cells.
(0.11 MB TIF)

Figure S6 Stable HCT116 BaxKO cells expressing Bcl-xL or Bcl-xL G138A were either grown in complete medium or starved for 6 h. Whole cell extracts were performed and 75 µg of proteins were separated on SDS-PAGE. Western blot was followed by immunodetection of Atg5-Atg12 conjugate.
(0.13 MB TIF)
