# Supplementary figures and images for: Differential Dependence on Beclin 1 for the Regulation of Pro-Survival Autophagy by Bcl-2 and Bcl-xL in HCT116 Colorectal Cancer Cells
Source: PLoS One. 2010 Jan 18;5(1):e8755. doi: 10.1371/journal.pone.0008755 (PMC2807451; doi:10.1371/journal.pone.0008755)

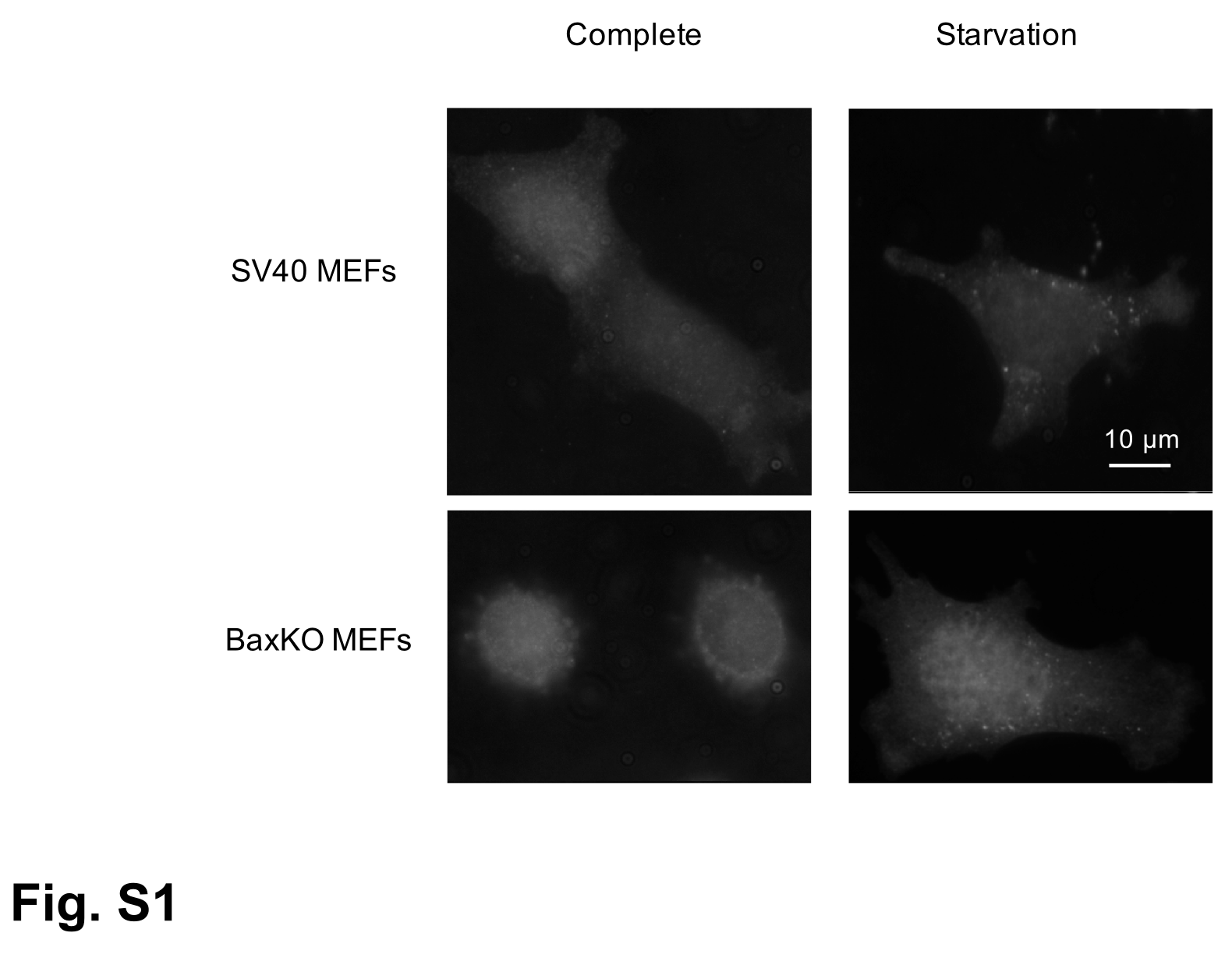

Supplement: Figure S1 — Mouse embryonic fibroblasts wild type (SV40) or (b) knocked-out for Bax (BaxKO) were transiently transfected using Fugene HD (Roche) with mCherryLC3. Cells were grown on gelatine-coated cover-slips, fixed in 4% paraformaldehyde for 40 mins, and washed. Cells were finally mounted with ProlongTM antifade polymerizing solution (Molecular Probes, Invitrogen) and observed on a Leica epifluorescence microscope. (0.45 MB TIF) [file pone.0008755.s001.tif]

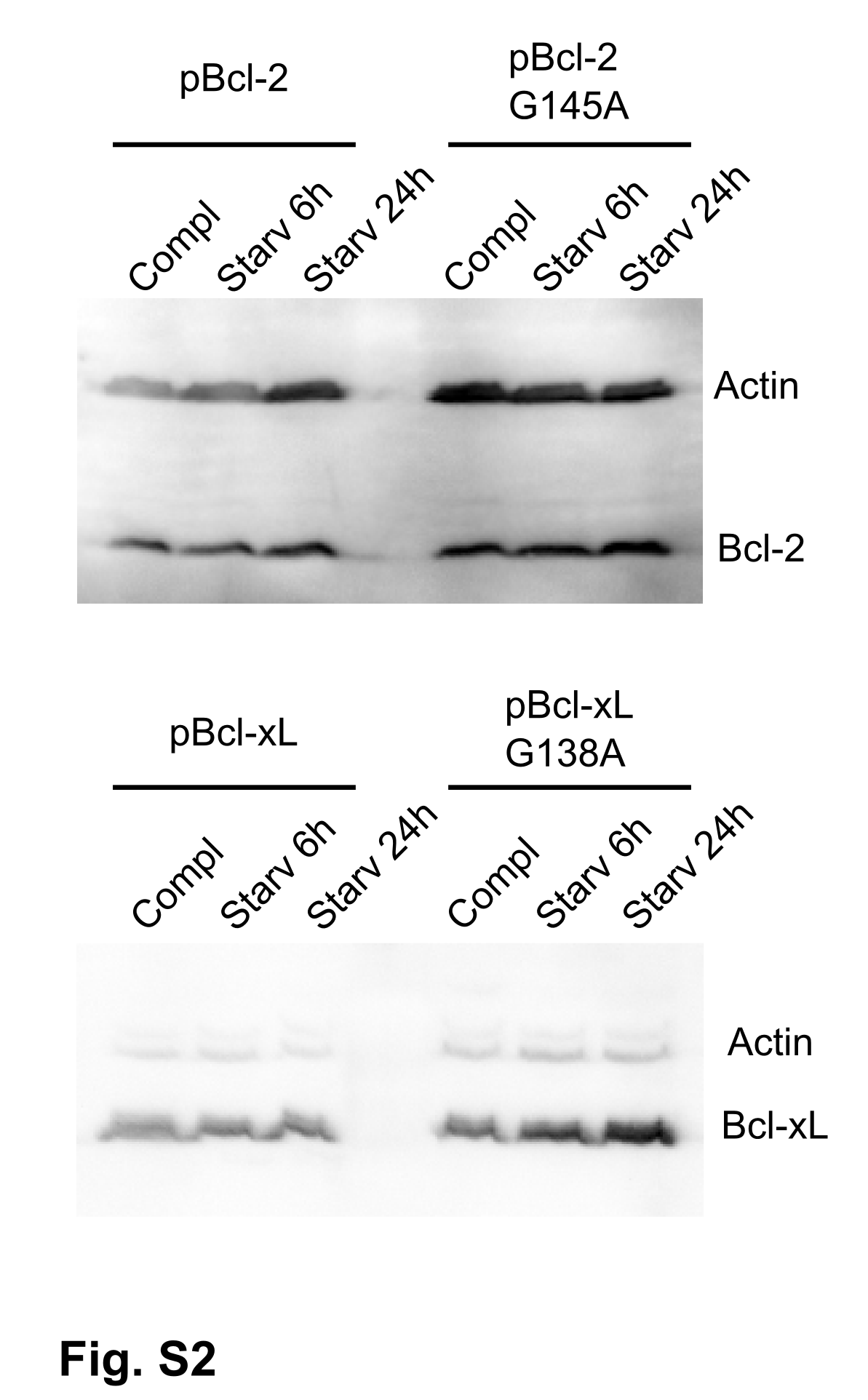

Supplement: Figure S2 — Stable HCT116 BaxKO cells expressing the indicated proteins of the Bcl-2 family were either grown in complete medium or starved for 6 or 24 h. Whole cell extracts were performed and 100 µg of proteins were separated by SDS-PAGE. Western blot was followed by immunodetection with actin as loading control or the indicated antibodies. (0.36 MB TIF) [file pone.0008755.s002.tif]

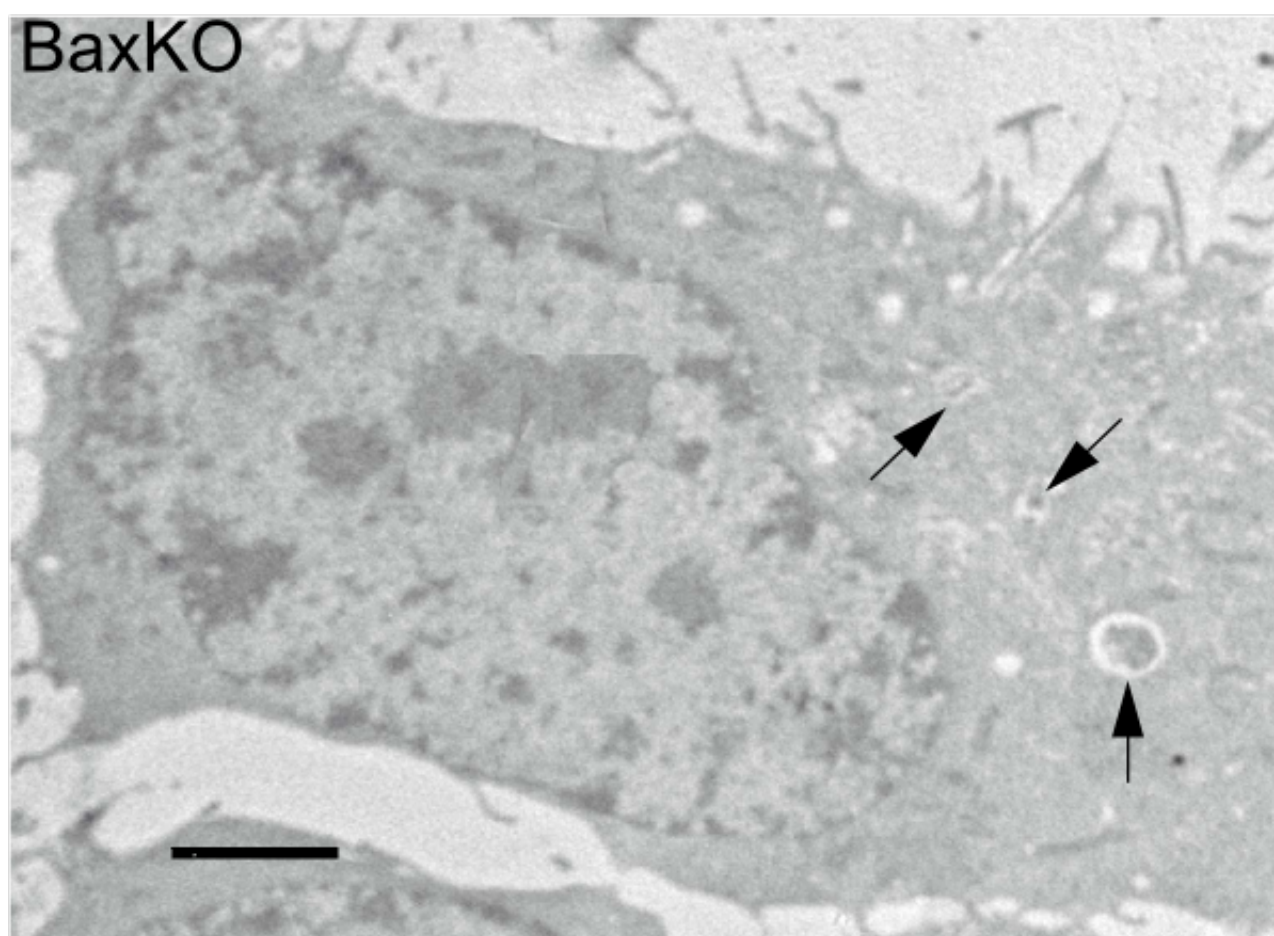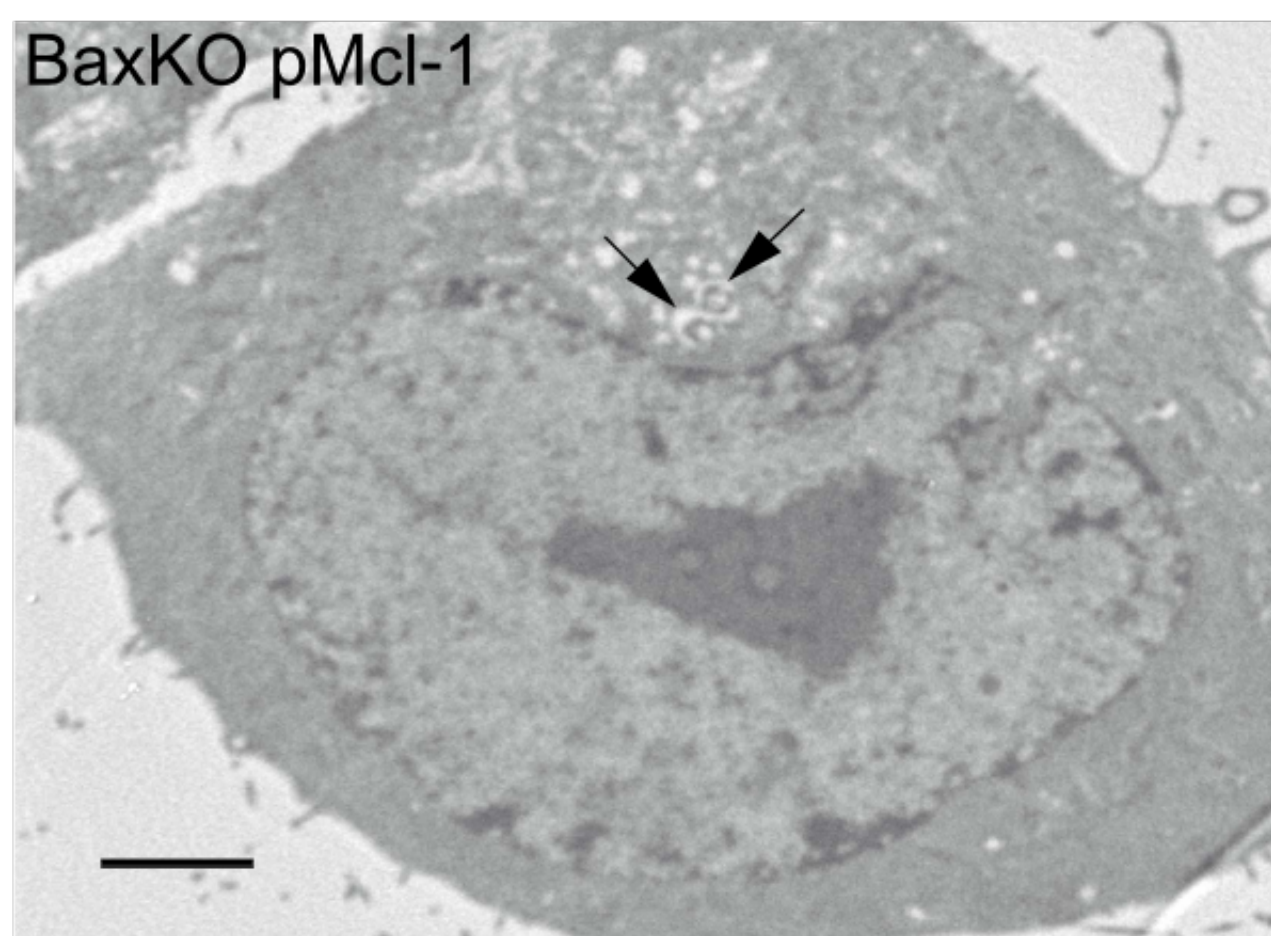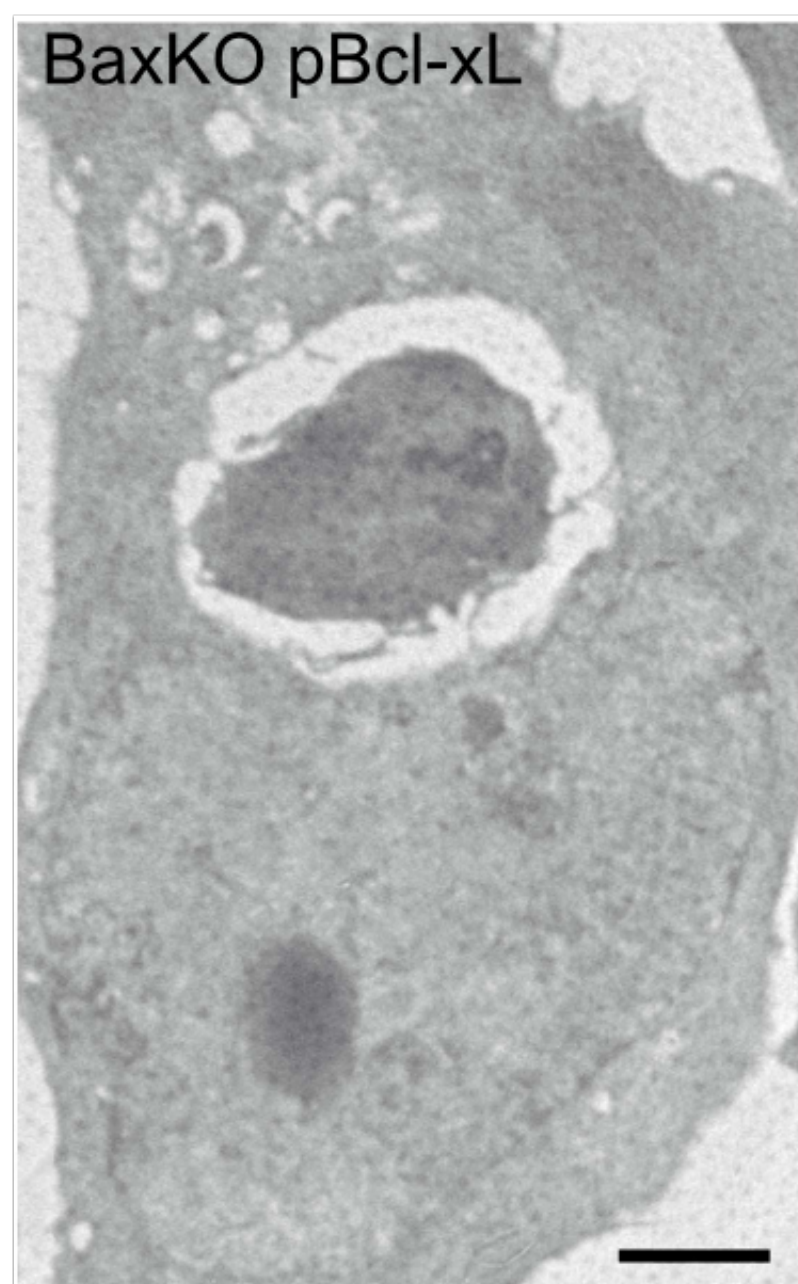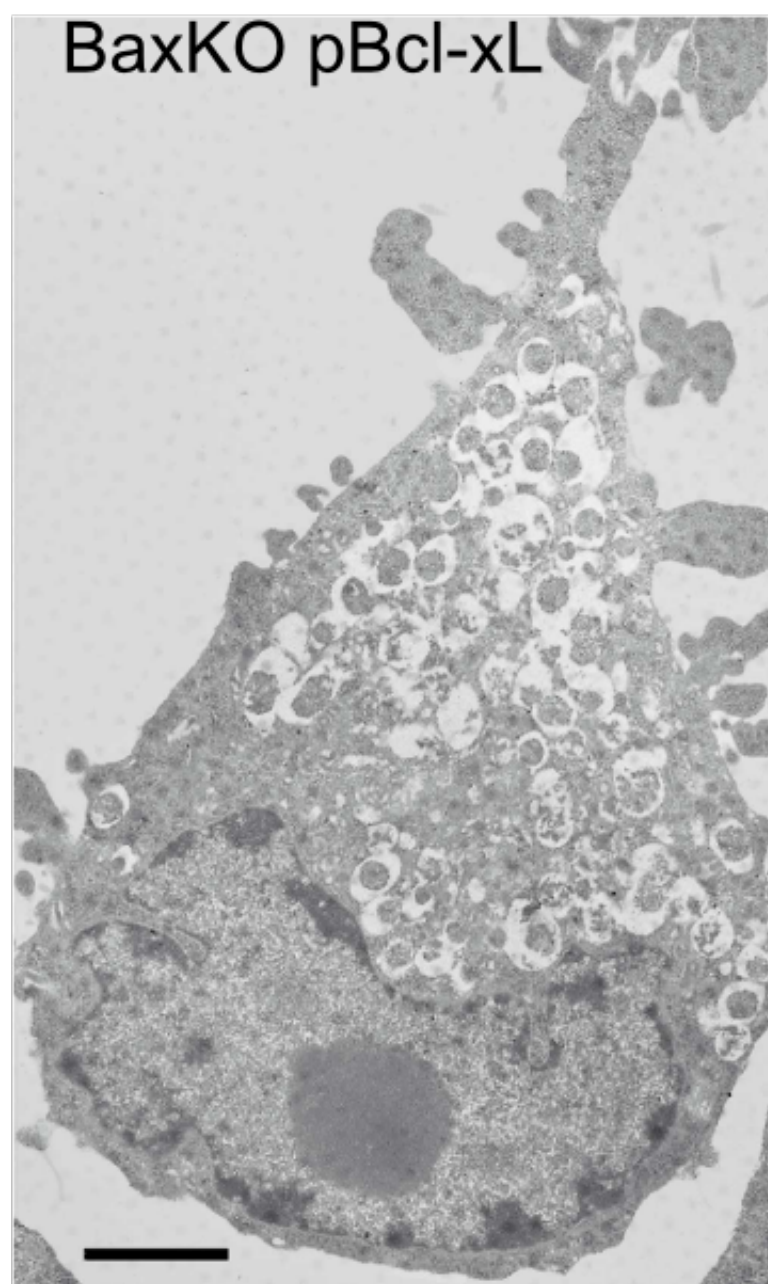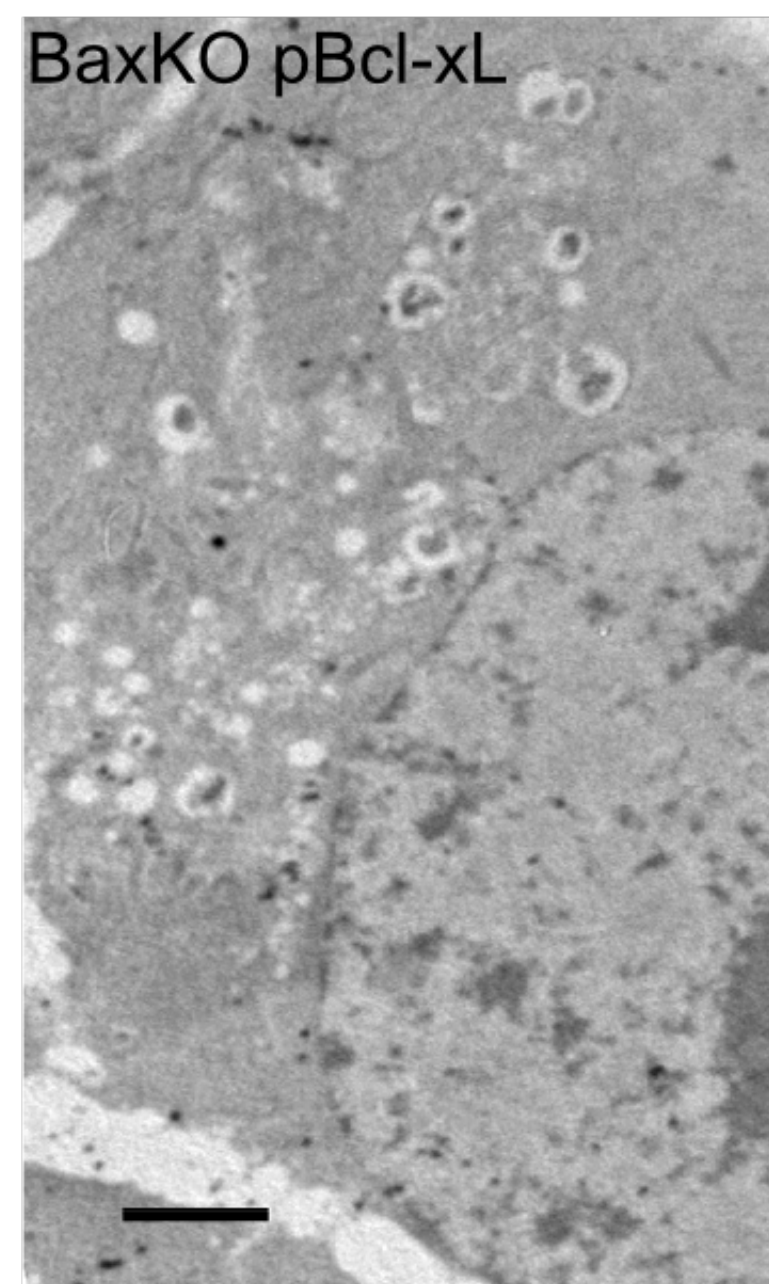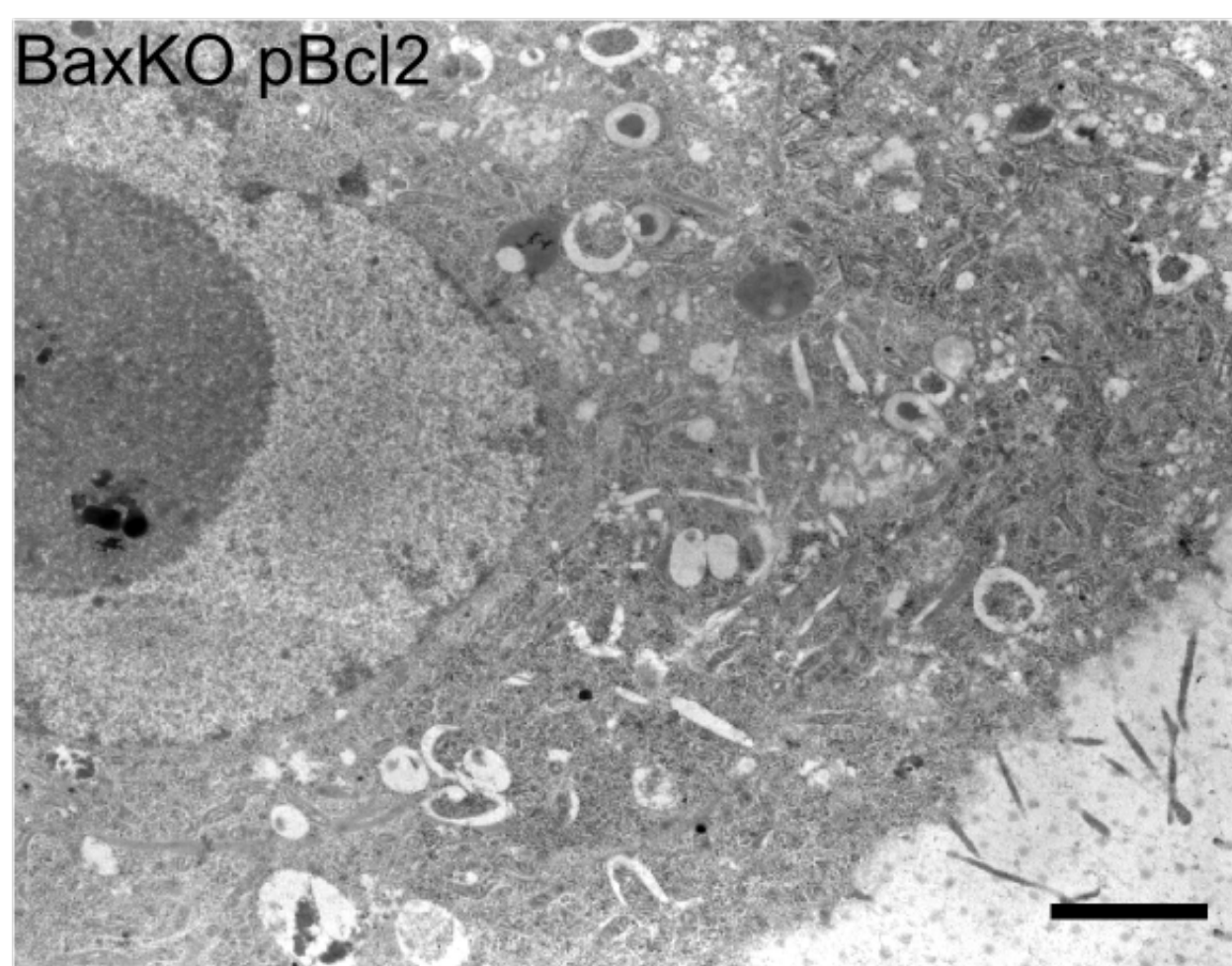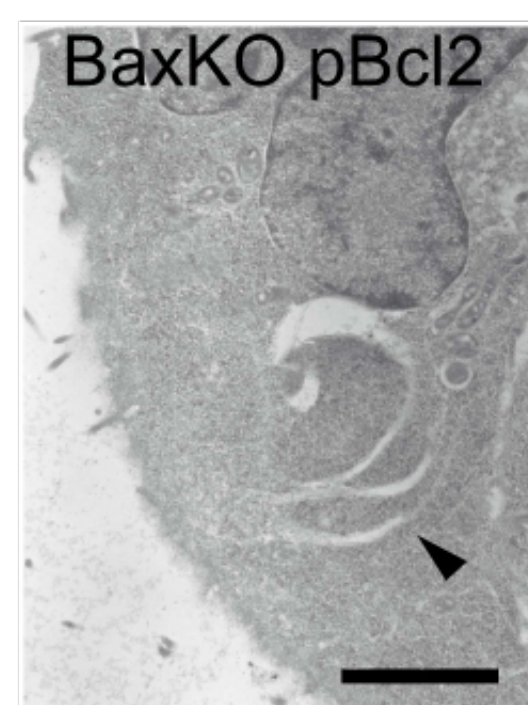

Fig. S3

2X zoom of TEM presented in Fig 3.

Supplement: Figure S3 — 2x zoom of TEM presented in Fig. 3. (1.82 MB PDF) [file pone.0008755.s003.pdf]

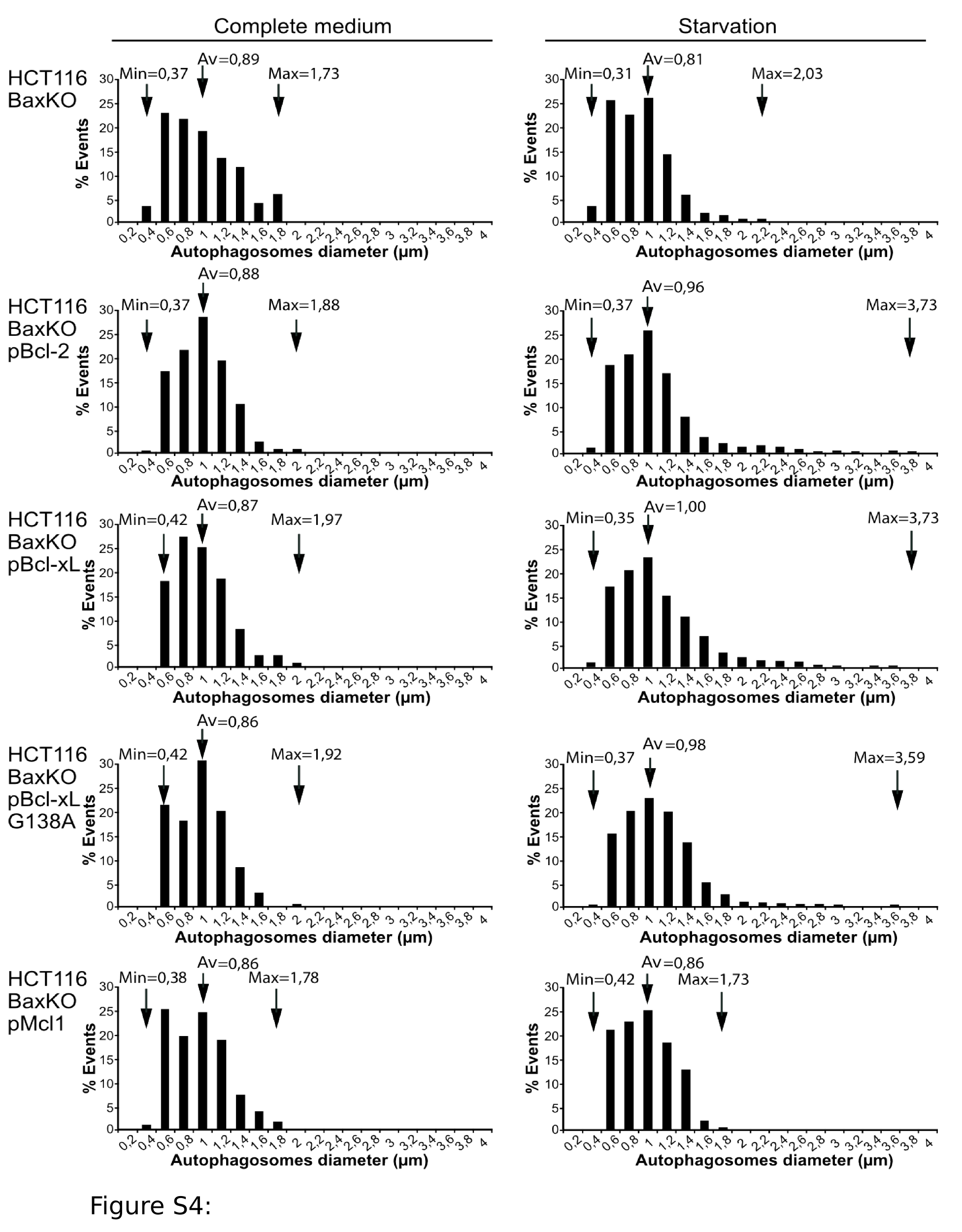

Supplement: Figure S4 — Stable HCT116 BaxKO cells expressing the indicated Bcl-2 family proteins were grown on gelatin-coated glass cover-slips to approximately 70% confluence either in complete medium or starved for 6 h. Cover-slips were washed with PBS, and incubated for 30 minutes with 200 µM MDH at 37°C in the dark then washed with PBS, and mounted for immediate observation under UV (λex = 359 nm) on a Leica DMLB microscope. Digital pictures were acquired with a Leica DC 300-F camera. Analyses of the images were done with a home-made program on a Leica Q550 imaging workstation. Computer-assisted analyses were performed on 200 to 400 cells to determine the diameter of all MDH-stained structures. The distribution of the diameters was plotted by intervals of 0,2 µm, and the frequency of occupation of these intervals labeled (% events). A Student test was applied for statistic analysis: in transfected cells, distributions under control conditions were not statistically different from that of untransfected cells (p = 0.42 for pBcl-2 cells, p = 0.24 for pBcl-xL cells, p = 0.2 for Bcl-xLG138A cells, and p = 0.17 for pMcl-1 cells). Within each cell line, the distribution under starved conditions was statistically different from their respective control distribution (p<0.0001) except for Mcl-1 (p<0.4). Within each cell line, distributions under starved conditions were statistically different from starved parental cells (p<0,00005). (0.78 MB TIF) [file pone.0008755.s004.tif]

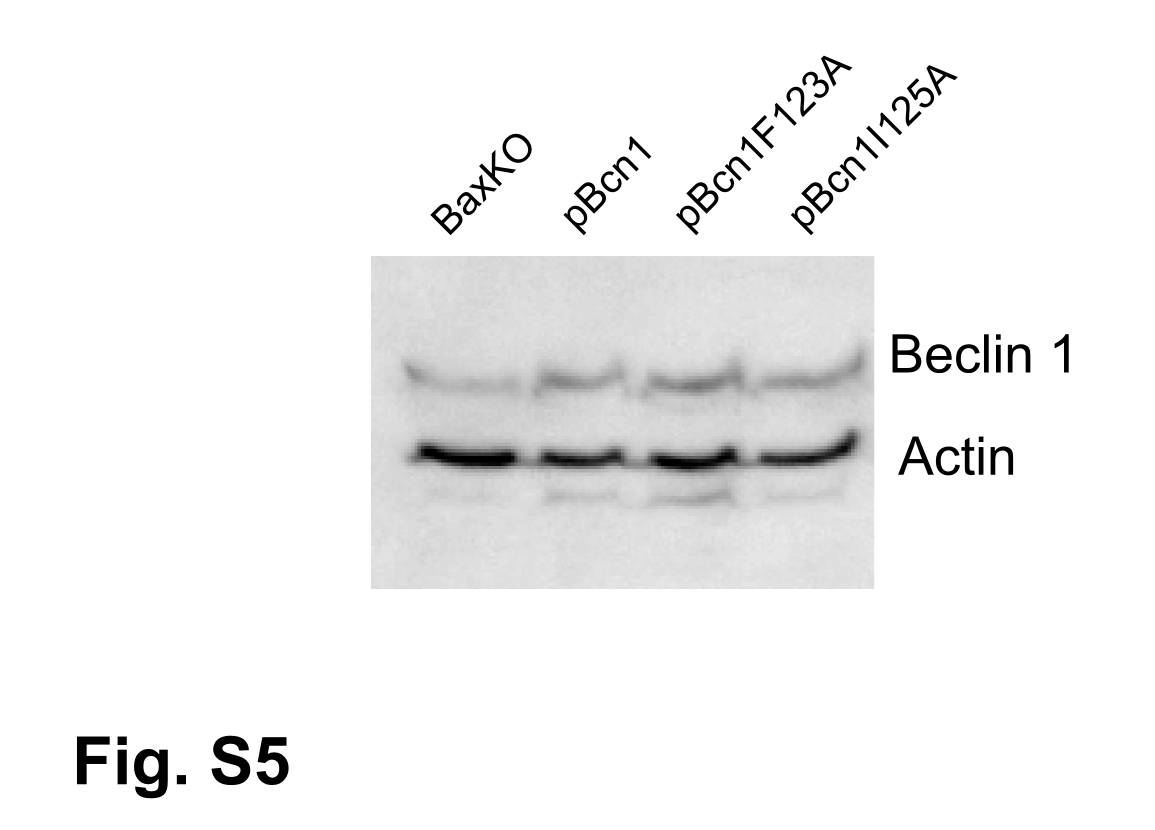

Supplement: Figure S5 — HCT116 BaxKO cells were transfected with plasmids encoding either Beclin 1 or the mutants Beclin 1 F123A or I125A. Whole cell extracts were performed and 100 µg of proteins were separated on SDS-PAGE. Western blot was followed by immunodetection of Beclin 1. Quantification with Image J software indicated that compared to endogenous level, the overexpression levels were 1.7 in transfected cells. (0.11 MB TIF) [file pone.0008755.s005.tif]

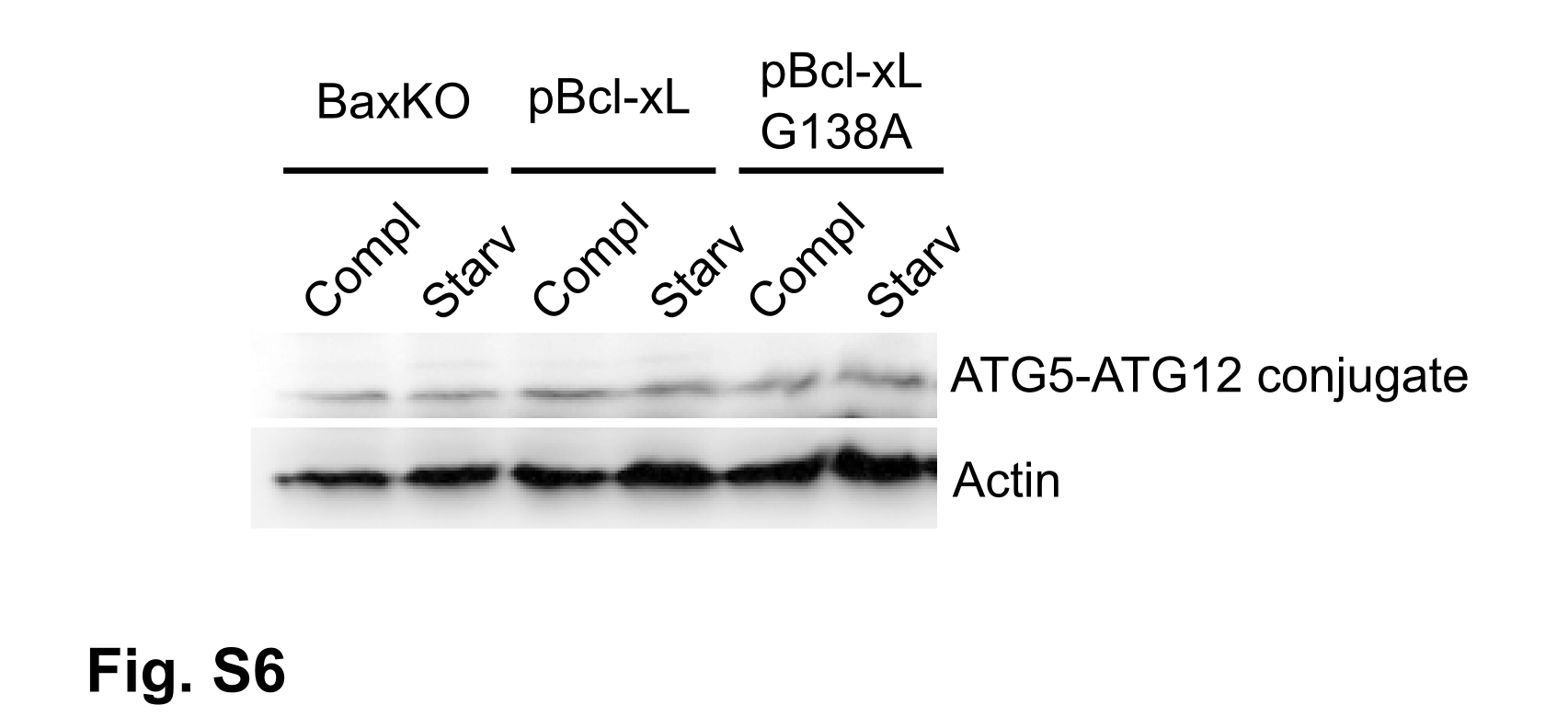

Supplement: Figure S6 — Stable HCT116 BaxKO cells expressing Bcl-xL or Bcl-xL G138A were either grown in complete medium or starved for 6 h. Whole cell extracts were performed and 75 µg of proteins were separated on SDS-PAGE. Western blot was followed by immunodetection of Atg5-Atg12 conjugate. (0.13 MB TIF) [file pone.0008755.s006.tif]
